# Lecture on Fever

**Published:** 1856-01

**Authors:** William Stokes


### DEPARTMENT OF SELECTIONS.

### LECTURE ON FEVER,

BY WILLIAM STOKES, M. D.

The study of the affections of the lungs in typhus leads us next to examine a class of cases in which a congestion, more or less severe, or it may be, a consolidation of the lung, takes place in connexion with the typhus state. You have already witnessed several cases of this kind. In some, there have only been signs of a congestive state, affecting a portion of one or both lungs; a state of stopping short of consolidation, and indicated by a crepitus with large bubbles, or a muco-crepitating rale, without much dullness, or the other signs of impermeability of the pulmonary tissue. In other instances, however, we have seen decided dullness occur; and, between these extremes, we meet with a number of cases varying in the degree, or amount, of the diseased action.

To these cases the general term of "typhoid pneumonia" has been given. But you will be convinced, when your experience has been enlarged, that under this term many different forms of disease have been classed; and it is very doubtful whether a true pneumonia is ever developed in the course of a typhus fever.—You will meet with the physical signs which attend pneumonia; but these, as you all must know, are insufficient to establish the existence of the disease; and even these very physical signs are seldom so well marked, so complete, as it were, as in simple inflammation of the lung. Nor again do they follow in the regular succession which we find in true pneumonia.

You know that I am not fond of fine drawn distinctions in disease, especially when these distinctions are based on some anatomical specialty, and do not lead to any differences in our principles of treatment; and we shall arrive at more practical results sooner by reviewing some of the more striking cases, I will not say of typhoid pneumonia, but of the acute asthenic diseases of the lung which tend to consolidate that organ. I mean, when using the terms "acute asthenic," to imply a disease which forms more or less rapidly, and is associated with, or secondary to, a condition of the system in which, with fever of some kind, we find evident signs of debility. We may recognize the following forms:—

1. Congestion with more or less consolidation in cases of what is called "diffuse inflammation," "erysipelateous inflammation," or, by some, "phlebitis."

2. Similar, or nearly similar conditions, (so far, at least, as we are taught by pathological anatomy) in cases of purulent absorption, with or without manifest phlebitis.

3. The intercurrent disease of the lung in cases of the eruptive fevers when they are of the low, putrid, or malignant type.

4. Congestions and semi-consolidations of the lung, as intercurrent affections in typhus fever.

5. Analogous conditions arising in the course of the non-maculated, or the so-called "typhoid fevers."

6. The disease occurring in connexion with delerium tremens from excess. In such cases, you will often find a group of asthenic local diseases, which are generally seated in the stomach, heart, the bronchial membrane, the parenchyma of the lung, and even the pleura. In some instances we find the patient has also typhus fever.

7. Rapid, extensive, and complete consolidation of the lung occurring in the course of a typhus fever. In some instances the patient dies asphyxiated; while in others, a portion of the pulmonary structures falls into sphacelus, and death takes place with the symptoms of acute gangrene of the lung.

Such are some of the more prominent cases which have been classed under the head of typhoid pneumonia. There is another, of which we have had many examples, and yet I do not wish you to take what I am going to say about it in any other way than in the light of suggestions. The case, as I said before, is by no means uncommon. The patient is attacked with the usual symptoms of typhus fever, and he comes into Hospital after two or three days' illness. There is nothing about him to make one think that his disease will not run the usual course of the epidemic of the day, and we are prepared to expect a fever of at least a fortnight's duration. On admission he may have no symptom which would call attention to his chest; but, as early in some cases as the beginning of the fourth, and in others the fifth day, it is discovered that the upper lobe of one lung is solid, or nearly so. The clavicle is quite dull on percussion, so is the scapular spine, and the dullness extends to the line of the mamma. This discovery has been so often made accidentally, that I am sure that many of such cases have passed unnoticed, at least, where the attendant is not well informed as to the insidious nature of typhus local diseases, and does not make it a practice and duty to examine daily, as far as he can, the condition of every organ.

But the most remarkable circumstance in these cases is, that the constitutional disease seems to be cut short. The expression of fever leaves the countenance; the peculiar color or hue of typhus disappears; the eye becomes bright and intelligent; the tongue clean; and the pulse comes down to a natural state.—And thus we have seen patients so altered in the course of twenty-four hours, that one had some difficulty in recognizing them. All the symptoms of typhus were gone, and nothing remained but the consolidation of the lung. And this, too, is not attended with any notable suffering. There may be a little cough, some dull pain, or an inability to lie on one side; but that is all. The respiration is scarcely, if at all, accelerated; in fact, it would seem that there was no irritation or excitement of the organ; and the case is another proof of how much less the sufferings in disease are connected with the mechanical than with the vital conditions of organs.

This local disease, too, is generally easily managed. A small blister or two, the application of the tincture of iodine, and the exhibition of a little hydriodate of potass, with or without a tonic, will remove it. Indeed, the cure is often so rapid, that I have thought that our remedies had little to do with the result. How are we to look at such cases? That they are not examples of inflammation of the lung is plain, and it appears probable that if this local disease had not occurred, the patient would have gone through the course of the fever of the day. Does it not seem as if the constitutional disease exhausted itself as it were, in the production of the local affection, just as, in certain cases of simple variola, we see the fever subside on the appearance of the pustule? I do not know whether such cases have been observed elsewhere, but of their existence we have had abundant proofs. It is worthy of remark, too, that when we compare these cases with the ordinary forms of typhus, attended with secondary disease of the lung, the local affection is here developed at an unusually early period; and it may be, that in the more protracted cases of fevers, the nature of which is to develop local affections, the periods of this development and of the cessation of the fever may also be coincident. We do not, however, find that this is so common as to establish a rule. Let us, assuming that these various cases are really examples of typhus with a secondary deposit, again compare them with the more ordinary forms of the disease, and we shall find that they want two important characteristics of the longer fevers; one, the successive production, or the simultaneous production of various local diseases; and the other, the occurrence of that secondary inflammation, or irritation of the parts in which the deposit takes place. That the latter circumstance is one of great weight in relation to the preventing or delaying of crisis, it is impossible to doubt. As to the case of successive, or simultaneous production of local diseases, this, at all events, marks a more severe and complicated disease.

Gentlemen, I will not here enter into the wide subject of crisis in fever; yet I may point out to you, as a matter well worthy of investigation, the possibility of the occurrence of crisis by other modes than those which are generally enumerated; thus we may have a crisis without sweating, or diuresis, or hemorrhage, or diarrhœa, but which takes place by an internal and silent change in the condition of an organ, and yet a change which will, or may, itself spontaneously disappear.

Here let me warn you against the error which so many fall into with respect to these various cases of disease of the lung, arising in the course of some form of constitutional disease of fever. They are usually set down as pneumonia; typhoid pneumonia by some. Now the name itself would be of little moment if its adoption did not lead to errors in practice. And, although it cannot be affirmed with certainty that in none of these cases is there pneumonia, yet we have good grounds for believing that in many of them, inflammation, as the term is commonly understood, is either absent from the first, or, if it occurs, that it is only secondary to a special lesion induced by some form of constitutional disease.

It is extremely difficult to present to you any well-defined classification of the various forms of diseases which have been described under the head of typhoid pneumonia, or to draw the line between simple asthenic inflammation of the lungs, and those conditions which have been described from an early period under the terms of bilious, putrid, or typhoid pneumonia. And observe when I make use of the term asthenic pneumonia, we refer more to the condition of the general system than to the activity or inactivity of the local disease. For, so far as local inflammatory action is concerned, there is abundant proof that it may originate and proceed with rapidity, and even with vehemence in the very last periods of life, so that the disease may be sthenic, *quoad* the local condition, and yet the case itself be asthenic in reference to the general state of the economy. Much of the confusion with regard to this subject has arisen from the circumstance, that too great weight was attached to the presence of certain physical signs which were taken as always indicating similar conditions. The succession of the signs of crepitus, dullness, cessation of vesicular breathing, and its replacement by bronchial respiration, has been admitted to indicate a simple pneumonia, in which the local disease is the principal pathological condition, and the fever only a secondary one. But it is certain that this train of pneumonia, or some modification of them, may occur under exactly opposite circumstances, the local disease being symptomatic of the fever, and not the fever of the local disease. And there is the strongest reason for believing that even though the mere anatomical condition of the lung in the two cases be similar, yet there is an essential, a vital difference, and that practically we cannot deal with the local disease in the latter case as if it were an original affection. This applies to all those cases of disease with the physical signs of pneumonia, which are secondary to any forms of fever, whether it be typhus or typhoid, whether it be variola or erysipelas, purulent poisoning of the blood, glanders, malignant scarlatina, or malignant measles. In these cases, even though the physical signs accurately correspond with those of the typical pneumonia, which, by the way is by no means always the case, we must believe that we are dealing with a special condition of parts, and a condition not only special, as compared with ordinary pneumonia, but having also another specialty—namely, that which is derived from the parent malady.

In the present state of our knowledge, gentlemen, we cannot declare that any special anatomico-pathological condition exists, by which we can distinguish these secondary diseases one from the other; but we may say this much, that practically they all appear to agree in being indicative of an asthenic state of the system, and that, therefore, the supervention of their physical signs at any period of those various diseases, must not be permitted to divert your attention from the general condition of the patient, or make you proceed to treat a case as one of sthenic pneumonia because it has some, or even all, the physical signs of that condition. Do not suppose that I am taking up your time unnecessarily by insisting on these points, for they lead us directly to deal with one of the greatest, if not the greatest and most widespread error in the practice of Medicine; namely, the treatment of all local, acute and febrile diseases as inflammations. Here is a group of acute, local, and febrile diseases, and not only this, but a set of cases exhibiting some or all the physical phenomena of acute pneumonia; and yet, if we subjected them to the ordinary routine treatment of inflammation, the worst consequences would almost certainly follow. You must learn to look at the antecedents and the accompanying general phenomena of these diseases, and set your face against the adoption of any treatment which is based on the doctrine that they are original inflammations. I believe that the erroneous views to which I allude, are, I am happy to say, every day becoming less and less frequent (thanks to our improved system of clinical instruction) and the independent spirit of investigation which now animates so many of our students. Notwithstanding, they are still too often acted upon, and over and over again, patients who have enough to contend with as the victims of some fell fever or other constitutional disease, are lost, or assisted to their death, by the adoption of a local or general anti-phlogistic treatment, in consequence of the physical signs of a pneumonia being discovered; their stimulants are withheld or withdrawn; tartar emetic, or mercury, or even blood-letting is rashly resorted to; and it often happens that, even though the physical signs of the pneumonia are removed or modified, the patient sinks from the combined effect of the original disease and the exhaustion produced by this ignorant and benighted treatment. I do not think that any of you will fall into these or similar errors after what I have so often said; it will, at least, not be my fault if you do.

Let us now consider what we may term the parenchymatous affections of the lung in fever, or, if you will, the typhus disease of the pulmonary structure. It may be stated generally, that whatever be the differences in the various cases of this affection in typhus, the local disease follows the general law of other lesions secondary to the fever; that is to say, it agrees with them in its mode of invasion, its latency in the earlier periods of its development, its frequent spontaneous retrocession, and lastly, in its pathological effects. It is quite true, that, as compared with the best marked examples of acute sthenic pneumonia, it is not wanting in any of the physical signs of that disease taken singly, but it is generally different from it in the order or arrangement, as it were, of these physical signs. And indeed, I think it is an extremely rare circumstance to observe in the course of a typhus fever the rise, progress, and retrocession of a pneumonia which has passed into hepatization, as we so continually see in ordinary cases of the disease. I have already drawn your attention to those curious cases of consolidation of the upper lobe of the lung; now, whether these be genuine examples of an arrested typhus or not, it is difficult to say, but their whole history and progress is very different from that of ordinary pneumonia; and I repeat, that there is nothing more rare than to see in the course of a typhus fever the regular succession of phenomena with which Laennec has made us so familiar, as indicating the several successive stages of an acute pneumonia occurring under what may be called its normal conditions.

The most common case is the occurrence, generally at an early period, especially in the maculated forms, and often at a later period in the non-maculated, or so-called typhoid fevers, of a well-marked crepitating rale in the lower lobes of one or both lungs; it is generally much more extensive and distinctly marked in one lung than the other. The amount of dullness is seldom very great, and we find the disease, as it were, to linger, and for days together to show no disposition either to produce solidity of the lung on the one hand, or to proceed to resolution on the other. The disease is often quite latent, and is only recognizable by careful physical examination; and its discovery, as you will readily understand from what I have said before, is sometimes an unfortunate circumstance for the patient. In the present state of our knowledge we must believe this condition of the organ to be either the result of a certain amount of typhus deposit into the lung, or of special state, an inflammation, if you will, which is, however, under the general law of the fever, partaking of its specific character, and capable of spontaneous retrocession. It is seldom attended by pain or by hæmoptysis, and it constantly exists without any important modification of the general symptoms of the case.

The second form of the disease is of a more serious character, and seems to be connected either with an original pyogenic disposition, itself secondary to typhus, or we may suppose that the typhoid deposit undergoes a rapid purulent transformation, so that in this way a condition of the lung is rapidly established, having some resemblance to the third stage of pneumonia, as described by Laennec. I have not myself seen a sufficient number of these cases to justify me in speaking very decidedly as to their physical signs; but I think I have seen enough of them to warrant me in believing that the course of the disease is different from that of ordinary suppurative pneumonia. We have not observed the intermediate stage of well-marked hepatization between that which is characterized by the occurrence of early rale on the one hand, and the signs of interstitial suppuration on the other. The complete dullness and the bronchial respiration which accompany the third stage of pneumonia, as described by Laennec, we have not observed; the physical signs being principally a persisting rale passing from a fine into a large crepitus, and semi-dullness on percussion. On dissection, the lung is found soft, friable, of a greyish-red color, but still very permeable to air, though infiltrated with purulent matter. It is as if the purulent secretion took place co-incidently with or immediately after the first or congestive stage. Some of the patients have had sweatings and a sanguinolent and somewhat sanious expectoration; but we have not hitherto observed the ordinary prune-juice sputa in these cases. I have seen this disease in connexion with purulent deposits in the neck, and posterior mediastinum, but it may occur without the formation of purulent matter in any situation other than the lung; it may supervene in the advanced periods of the case, and at a time when the patient seems about to recover; or it may come on much earlier, and when the skin is thickly covered with the petechial eruption. The last case is the most formidable; though one attended with the greatest danger, the disease is, however, not always fatal, and we have had several cases in which recovery took place. I need not say, that they were all treated upon a tonic and stimulating plan, in addition to which we employed dry cupping and blisters.

The last case of which I shall speak to-day is by far the most formidable of the pulmonary affections of typhus; it is characterized by the sudden, complete, and singularly extensive consolidation of the lung. In the course of twenty-four, or even, sometimes, of twelve hours, the most extensive and complete dullness may be produced in a lung which had been previously free from physical signs, or, at most, had only exhibited some of the ordinary bronchial rales of typhus. We have thus the signs of complete hepatization, unpreceded by the crepitating rale; the disease begins by consolidation, and then one of two results follows; either the patient dies speedily, generally with loose rales in the opposite lung, combined with tracheal effusion; or, after a day or two, he begins to expectorate a horribly fetid matter, and we discover by the stethoscope that a large cavity has fromed in the lung. This is a true gangrenous cavity formed in the very centre of the solidified mass, and the disease has a close pathological analogy to the process of acute mortification, which has been described, as occurring in some of the worst cases of the typhus disease of the intestinal glands.

Let us now pass in review the circumstances in which these forms of disease occur; for when we compare them with the ordinary conditions of acute primary pneumonia, we cannot but admit that they indicate a lesion of a very different nature.

In the first place, the physical signs are preceded by fever; and it may not be until many days have elapsed that the symptoms of lung affection, as it were, spontaneously arise. 2ndly, the fever is obviously an essential fever; it may occur with or without petechiæ, and other complications may or may not be present. 3rdly, the disease sets in without any apparent external exciting cause. 4thly, when the purulent form is observed, it appears to be, as it were, the second stage of the affection; and I may here remark, that, on dissection, we rarely, if ever, find what is termed concrete or nonconcrete purulent matter. Lastly, the invasion of one form of the affection may be sudden, and the signs of extensive and complete consolidation be among its earliest phenomena. It is in this case, too, that, if time be allowed, large eschars forming cavities, which may communicate to the bronchial tubes, are liable to occur.

I may remark here, that in two cases of this rapid consolidation, the gangrenous eschar did not communicate with the bronchial tubes. One was a case of typhus, in a man who had long suffered from gangrene of the opposite lung; the other occurred in a case of what is termed the erysipelatous or diffuse inflammation.

CONTINUED IN NEXT NUMBER.

